# Requirements of a Group Intervention for Adolescents with Internet Gaming Disorder in a Clinical Setting: A Qualitative Interview Study

**DOI:** 10.3390/ijerph18157813

**Published:** 2021-07-23

**Authors:** Lisa Marie Wendt, Maria Isabella Austermann, Hans-Jürgen Rumpf, Rainer Thomasius, Kerstin Paschke

**Affiliations:** 1 German Center for Addiction Research in Childhood and Adolescence (DZSKJ), University Medical Center Hamburg-Eppendorf (UKE), Martinistraße 52, 20246 Hamburg, Germany; li.wendt@student.uni-luebeck.de (L.M.W.); ma.austermann@uke.de (M.I.A.); thomasius@uke.de (R.T.); 2 Department of Psychiatry and Psychotherapy, University of Lübeck, 23562 Lübeck, Germany; Hans-Juergen.Rumpf@uksh.de

**Keywords:** internet gaming disorder, addiction, problematic gaming, group therapy, adolescents, qualitative interview study

## Abstract

Internet gaming disorder (IGD) has become an important health concern in a significant proportion of adolescents. Intervention studies in this age group are scarce, mostly follow quantitative designs, and rarely consider adolescents’ experiences. This study aimed to evaluate the requirements for a group therapy program for adolescents with IGD. A qualitative interview study was conducted in a German clinic for addictive disorders in childhood and adolescence with nine participants (seven IGD patients (12–18 years, M = 15.86, SD = 1.95) and two psychotherapists). The semi-structured interviews addressed helpful contents, general conditions, and suggestions for alterations for an effective group intervention. Data were analyzed using content structuring qualitative analysis. Patient interview data resulted in 234 codings with eight main categories. Expert interview data yielded 151 codings with six main categories. The following treatment components were described as effective by the participants and experts: psychoeducation, emotion management, behavior analysis and modification, social skills training, parent participation, and relapse prevention. Additionally, adolescents emphasized the importance of group functionality for coherence, feedback and rewards, content presentation, physical activity and fun. The results are a valuable addition to findings from quantitative studies on IGD interventions and an interesting starting point for further representative studies.

## 1. Introduction

In the last two decades, the availability and the use of digital games has increased immensely [1,2]. Especially for young people, gaming has become an attractive leisure activity. With a lack of alternative activities, the time spent gaming has significantly increased among adolescents under lockdown conditions in Germany [3]. Digital games are created to generate high user engagement and to address specific interests of children and adolescents [4], such as discovering and creating new worlds, accomplishing complex tasks, and testing social roles in a virtual context [5]. However, intrapersonal and interpersonal risk factors can lead to an uncontrollable use of digital games that results in personal, social, and academic difficulties [6]. Adolescents are considered to be especially at risk, as their brain is still in the process of intense maturing and particularly vulnerable to external influences [4]. Accordingly, between 1.2% and 5.9% of adolescents in Europe, Asia, and Australia use digital games in an addictive manner [7]. A major step towards a uniformed and internationally agreed concept to describe problematic use patterns was taken by including criteria for internet gaming disorder (IGD) in the appendix of the Statistical Manual of Mental Disorders 5 (DSM-5) in 2013 [8]. Accordingly, an IGD is assumed if more than four out of nine criteria referring to gaming on the internet, or on any electronic device have been met in the past 12 months: (1) preoccupation with gaming, (2) withdrawal when not playing, (3) tolerance, (4) unsuccessful attempts to reduce or stop gaming, (5) giving up other activities, (6) continuation of gaming despite problems, (7) deceiving or covering up gaming, (8) gaming to escape adverse moods, and (9) risking or losing relationships or career opportunities due of excessive gaming. Furthermore, gaming disorder (GD) will be included in the 11th revision of the International Classification of Diseases (ICD-11) produced by the World Health Organization (WHO), as a new diagnosis coming into effect in January 2022 [9].

IGD in adolescence is often accompanied by dysfunctional emotion regulation strategies [10], as well as emotional and behavioral problems [11]. Affected adolescents often neglect school and private responsibilities, spend less time with friends and in leisure activities in the real world, and show increasing conflicts in family interactions [6,7,12,13]. Moreover, IGD is often accompanied by mental comorbidities such as depression [14], attention-deficit hyperactivity disorder (ADHD) [15], autism spectrum disorder [16], oppositional defiant and dissociative disorder [17] and (social) anxiety [18].

With regard to the clinical relevance of this new diagnosis, health care professionals call for effective treatment strategies. However, the number of systematically evaluated programs for children and adolescents in clinical settings is still limited.

Among all approaches for the treatment of IGD in adolescents, programs based on cognitive behavioral therapy (CBT) are the most common and best investigated ones [19,20,21]. These often include group therapy settings since, besides being economic, they could be shown to be effective in the treatment of mental disorders, such as obsessive-compulsive [22] and anxiety disorders [23] in adults, and depression in adolescents [24].

Some programs are complemented with other interventions, such as a psychoeducation group for parents [25,26] or the involvement of the patients’ parents in therapy [26,27,28]. The co-treatment of comorbidities is rarely focused on [12]. Evaluation studies are usually based on a quantitative study design and have mainly been conducted in the Asian region. Within these, treatment effectiveness is often defined by a significant reduction of digital media use times and addictive symptoms [6]. Moreover, only very few randomized controlled trial studies exist [29,30,31]. In general, CBT has been shown to be an effective treatment [20,21]. Alternative treatment programs, such as virtual reality therapy (VRT), might be considered effective regarding reduced problematic gaming symptoms but could not be shown to be superior to CBT [29]. Li and Wang [30] showed that CBT achieved significantly higher treatment effects than basic counseling on total Cognitive Distortions Scale (CDC) scores, all-or-nothing thinking scores, and online comfort scores. Marginally better treatment outcomes for CBT were found for short-term thinking scores. Nielsen et al. [31] showed that multidimensional family therapy (MDFT) keeps families in treatment with more success than family therapy as usual (FTAU). Treatment was completed in all MDFT cases and 70% of FTAU cases. However, a potential heterogeneity of CBT approaches regarding contents and settings, the lack of RCT studies, cultural sample differences, different underlying concepts of IGD, inconsistent use of instruments to measure the primary outcome, low case numbers, and/or high drop-out rates make it difficult to draw conclusions on the general effectivity of treatments and to derive recommendations for clinical practice.

Given the early state of research on this complex and novel disorder, individual experiences of patients and experienced practitioners are of high relevance for the development and the evaluation of treatment programs. To the best of our knowledge, until today only one qualitative study has been published that investigated adolescents’ perspectives on useful components and mechanisms in the treatment of problematic gaming behavior by semi-structured interviews [32]. The sample of Sim et al. consisted of 10 boys from 13 to 18 years and 11 parents who had participated in the Cyber Wellness Enrichment Program (CWEP) within two years before the interview [32]. The authors do not give information on comorbidities, diagnostic criteria, and the severity of the problematic behavior. The CWEP is a 3-month theoretically based preventive and treatment program offered by a non-governmental organization for adolescents in Singapore which includes individual, family, and group elements covering time management, social support, and family-based strategies. According to the study results, counselors played an important role in dealing with gaming-related issues in individual, family, and group settings. For example, role-modeling healthy video game habits, developing alternative activities, and addressing parental needs were seen as helpful.

We are aware of only one study that has addressed clinical professionals’ perspectives on the treatment of adolescents with IGD. Lo et al. [33] conducted a qualitative interview study on the role of family involvement in the therapy of adolescents with internet addiction (including IGD) in Hong Kong. The results suggest that family-based interventions facilitate the treatment process and encourage positive treatment outcomes by, e.g., supporting effective communication within the family.

To date, no qualitative study has been available on treatment needs for adolescents diagnosed with IGD in full or partial inpatient care in a group therapy setting. Moreover, no interview study has been conducted in Europe to examine experiences, specific demands, and needs for IGD interventions in adolescents. The current study aimed to fill these gaps by conducting a qualitative investigation of young IGD patients and clinical experts to contribute to a better understanding of treatment requirements for this new disorder.

## 2. Materials and Methods

### 2.1. IGD Group Therapy Program

The IGD group intervention applied in this study was an adaption of a manualized German CBT-based program (Lust for Life instead of Online Escape (ger. Lebenslust statt Onlineflucht)), designed to meet the requirements of the local (partial) inpatient setting including heterogeneous and changing group constellations, a high severity of individual symptoms, comorbidities, and an average therapy duration of 12 weeks.

The original weekly program comprises eight modules of 90 min each for four to eight patients and was developed at the German Center for Addiction Research in Childhood and Adolescence (DZSKJ) at the University Medical Center Hamburg-Eppendorf (UKE) [34]. Program goals are to provide psychoeducation about IGD; to critically reflect on and reduce individual problematic gaming behavior; to strengthen change motivation; to build up and enhance social skills; to support finding alternative leisure activities and functional emotion regulation strategies; and relapse prevention. These are addressed within the eight modules (translation by the authors):

1. “Program start and motivation of participants”.
2. “Development of a disturbance model”.
3. “Role of own feelings and individual goal setting”.
4. “Development of control behavior and compensation mechanisms”.
5. “Importance and development of relationships”.
6. “Interim review of achievements”.
7. “Relapse prevention and dealing with social pressure”.
8. “Conclusion and farewell”.

A first evaluation study in a quasi-experimental pre-post design with 18 adolescents found a significant reduction of problematic Internet use patterns and reduced average hours of use after program completion [35].

The adaption for the inpatient setting was realized by specialists of the DZSKJ and in cooperation with creators of the original program. This included, e.g., a reduction of the duration of one group therapy session to a maximum of 60 min, group sizes of no more than five patients, a focus on IGD, a simplified and age-appropriate presentation of contents, and a higher number of repetitions of theoretical information. Moreover, patients are asked to keep a diary of their digital media use, non-digital leisure activities, and their mood. The patients were professionally introduced to the IGD group (setting and content) before and in more detail within the first session they attended. The IGD program was integrated in the regular local inpatient and day-clinic (partial inpatient) treatment for adolescents with substance-related and behavioral addictions and mental comorbidities. This included individual psychotherapy sessions (once to twice a week), psychopharmacological medication if indicated, weekly cross-disorder group psychotherapies (open-topic group, groups on emotion regulation and social competence), occupational and sports therapy, adjacent experiential and individual pedagogical offers, clinical school classes, family support by IGD-specific parental therapeutic groups, individual family therapy sessions, and social counselling. The day clinic offers 8 and the ward 13 treatment places and is part of the Department of Child and Adolescent Psychiatry and Psychotherapy at the UKE.

### 2.2. Participants and Procedure

The study sample included seven patients and two clinical experts (*N* = 9). Thus, the sample size was in line with the recommendations for qualitative studies [36,37]. The sample was recruited from the day clinic and the ward for addictions in childhood and adolescence at the UKE between June 2020 and December 2020. The two experts were female psychological (child and adolescent) psychotherapists at the UKE with more than a decade of working experience with young addiction patients each. Both experts were specialized in the treatment of IGD in the (partial) inpatient and outpatient setting. Patients were included in the study when they were diagnosed with an IGD, which is coded under F63.8 (other habit and impulse disorders) in the ICD-10 [38]. They must have participated in the IGD group program for at least four weeks to ensure a sufficient program experience. Moreover, the interviewees needed to have the ability to reflect on their own experiences to actively participate in the interview dialogues. According to the evaluation of their therapists, 5 of 12 patients could not be considered for the interview (e.g., due to symptom denial and insufficient therapy motivation at the time of the study or due to serious comorbid symptoms including lack of energy, depressed mood, and severe concentration deficits). Before the interview patients were asked whether they agree to participate. They were informed that their participation was voluntary and could be revoked at any time.

The patients’ age at the time of the treatment ranged from 12 to 18 years (*M* = 15.86, *SD* = 1.95). In line with the gender-dependent IGD prevalence [7], the sample consisted of more boys (*N* = 6) than girls (*N* = 1). All patients had comorbid mental disorders, with ADHD and affective disorders being the most common ones. Of the seven patients, two had been discharged from the day clinic 14 and 28 months before the interview and were contacted via telephone. The other five patients had been in ongoing treatment for 4.8 weeks (*M* = 4.8, *SD* = 1.6) at the time of the interview (day clinic: *N* = 3; inpatient: *N* = 2). For more information on the sample, please refer to Table 1.

### 2.3. Interview Conduction

In order to understand which requirements a group therapy program for adolescents with IGD should fulfill and how the current program is perceived, two semi-structured interview guidelines for the affected adolescents and the clinical experts were planned and developed by the authors based on their clinical and scientific expertise on IGD in adolescence. Extracts from the interview guide are shown in Table 2. The guidelines for the adolescents included 16 questions, which can be assigned to five main topics: 1. current state of knowledge about psychoeducational contents and individual pathological gaming pattern, 2. effective, helpful, and less helpful therapy contents, 3. suggestions for program alterations and additions, 4. general conditions of therapy (group setting, content presentation, task processing), 5. handling of homework. The guidelines for the expert interviews comprised 13 questions. They can be assigned to four categories, similar to those of the patients’ interviews: 1. requirements for the participants and the leader of the therapy, 2. general conditions of the therapy, 3. effective and particularly demanding contents of the therapy, 4. form of presentation of the contents. The interview guides are attached in Appendix A and Appendix B.

All interviews were carried out in German. Since the interviews took place during the coronavirus SARS-CoV-2 (COVID-19) pandemic, they were held face-to-face in compliance with the hygiene guidelines or by telephone (with four patients). On average, an interview lasted 22 min (*M* = 21.6, *min* = 14, *max* = 30, *SD* = 5.1). The interviewer was a psychologist and not part of the treatment team of the patients. Before the interviews began, the interviewer gave a brief introduction explaining the general conditions of the study. This included the voluntary participation, content and aim of the study, the permission to record the interview for the transcript, and the anonymity of stored data. The interview guidelines were followed chronologically. In-depth questions were added if appropriate.

### 2.4. Analysis of the Data Material

The interviews were transcribed anonymously between 17 August 2020 and 18 December 2020 and analyzed using the program MAXQDA 2020 (VERBI GmbH, Berlin, Germany). The data were analyzed separately for the interviews with patients and experts using qualitative content analysis (QCA) with a category-based method developed by Kuckartz [39].

First, main categories were developed directly from the interview guidelines in the form of deductive category generation. After testing the applicability of the deductively developed categories, the category system was further developed and differentiated directly from the material after inductive category formation [39]. The categories were thus formed deductively-inductively. To increase intercoder reliability, the developed category systems were validated with an independent psychologist. Based on the initial category system developed, the first coding process of the entire material was carried out. Care was taken to ensure that each coding unit addressed only one category. Text passages that were ambiguous were excluded from the analysis and interpretation. The final differentiated category system was assigned to the coded text passages. For this purpose, the material was completely reanalyzed [39].

## 3. Results

### 3.1. Interviews with Patients

The analysis of the patient interviews resulted in 234 codings, which could be classified into eight main categories. Table 3 shows the main and subcategories with selected anchor examples and their reference. In the following, the results of the categories are presented.

#### 3.1.1. Patients’ Characteristics

The first main category on the characteristics of problematic gaming behavior contained 27 codings and could be divided into three subcategories from the data material:

1. Escape;
2. Neglect;
3. Loss of control.

All patients reported that they use gaming mainly to escape from stress, and/or to cope with negative emotions, and/or problems at school or within the family. Five patients described the neglect of school or family obligations as well as non-digital leisure activities as a negative effect of their gaming patterns. In addition, two patients emphasized regulation of gaming time as particularly difficult.

#### 3.1.2. Functional Aspects of Gaming

Besides problem behavior, two patients also stated positive and functional effects of gaming. These are summarized in the second main category with four codings. They described the digital gaming world as a safe and comfortable place to try out different roles and to undertake positive, interpersonal experiences that would not be available in real life.

#### 3.1.3. General Setting of the Group Therapy

The third main category included 54 codings that were classified into three subcategories with minor categories. Patients emphasized the role of the group therapy setting as comparably important to the therapy content.

1. Requirements for a functional group setting
  - Respectful interaction between group members;
  - Mutual support of group members;
  - Stable group constellations;
    - Difficulty with unstable group constellation;
    - No difficulty with unstable group constellation;
    - Positive group atmosphere;
2. Working on tasks;
3. Presentation of contents.

Four patients said that they had difficulty with changing group constellations. They found it more difficult to be open to new group members. Other patients described problems with the composition of the group mixing patients of the day clinic and inpatients. This contrasted with the statements of two patients who had no difficulties with an inconstant group constellation.

Four patients stated that getting to know new patients and having fun in the group were important for a confident group atmosphere.

Moreover, it became apparent that in a group setting it can be challenging to meet the individual preferences of the patients when it comes to the accomplishment of tasks and the presentation of therapy contents. Five of the seven patients describe themselves as visual learners and would have preferred a thoroughly visual presentation of the content.

#### 3.1.4. Effective Treatment Factors

The patients’ statements on effective or helpful factors in treatment could be classified into three subcategories with a total of 15 codings:

1. Problem identification and discussion;
2. Structure of everyday life;
3. Group cohesion.

In the three subcategories, six patients reported that they found it helpful to identify and discuss their individual problems in therapy, to structure their daily lives, to share experiences with other affected people, and to make progress together with the group.

#### 3.1.5. Experiences with Group Therapy Contents

The fifth main category comprised 53 codings and included the patients’ experiences with the contents of the group therapy. These could be classified into five subcategories with a total of eleven further subcategories:

1. Level of knowledge about psychoeducative contents;
2. Helpful group content;
  - Psychoeducation;
  - Addressing individual problematic behavior;
  - Fun;
  - Competent group leaders;
3. Interesting group content;
  - Mechanisms of IGD and regaining control over gaming;
  - Conception and mechanisms of games;
4. Less helpful group content;
  - Contents without personal reference;
  - Content without gaming reference;
  - Redundant content (with regard to other attended therapy groups);
5. Challenging group content.

All of the seven patients reported that they feel well educated in terms of IGD after having attended the group program. Thus, the psychoeducational content of the applied program seems to satisfactorily provide knowledge about the disorder.

However, four patients reported that they already had some IGD-specific knowledge before having started the program. Five patients named different aspects to be helpful in IGD psychoeducation. These included knowledge about causes and triggers of problematic gaming patterns, awareness of negative effects of gaming and learning how to individually deal with IGD. Three patients reported that it had been helpful for them to reflect on their individual problems and to be supported by finding alternatives to gaming. Moreover, having fun in therapy and believing the group leaders to be competent were mentioned as helpful by many patients.

Three interviewees named the exchange about mechanisms of IGD as particularly interesting as a tool to regain better control over gaming. Furthermore, understanding professional conception and mechanisms of games to create strong user attachment was reported as particularly fascinating.

According to the interviewed adolescents, contents would not be experienced as helpful if they seemed redundant to other therapy groups the patients attended, did not obviously relate to gaming, or could not be connected to the individual situation.

The first sessions of the group program were described as especially demanding. Two adolescents reported that they had difficulties in adapting to the requirements of the clinical setting at the beginning of their (partial) inpatient therapy in general.

#### 3.1.6. Increase and Maintenance Motivation for Group Therapy

The sixth main category included four subcategories with a total of 14 codings:

1. Feedback on individual progress;
2. Motivation through meaningful others;
3. Positive reinforcement;
4. Structuring of group therapy sessions.

The first three subcategories reflected that motivation was most enhanced and sustained by feedback on individual progress, rewards for progress, and through meaningful interactions with others, especially peers. Five patients described how their motivation could be maintained by the structure of the group therapy sessions. Above all, fun and sufficient physical activity in the sessions were claimed to be important motivational factors by several patients.

#### 3.1.7. Therapeutic Diary and Homework

Statements related to patients’ experiences with homework were categorized into six subcategories with a total of 24 codings in the seventh main category:

1. Motivational aspects for regular filling in of the therapeutic diary;
  - Self-responsibility;
  - Professional support;
  - Diary structure;
2. Volume of homework;
3. Optional homework.

A diary on daily gaming times has a key role in supporting self-monitoring and reflection in the group program the patients attended. The first subcategory contained statements on three subcategories on factors to increase motivation for regular diary completion. Three patients emphasized self-responsibility for the accomplishment of this recurring task. In addition, three patients argued for a need of support by therapeutic staff. In the third subcategory, two patients mentioned that they would be more motivated to keep the diary if it was easier and more appealing to fill it out.

Five of the patients expressed that extensive homework (e.g., tasks in addition to the diary) had been demotivating to them. In contrast, one patient said that optional homework would help him to fight boredom.

#### 3.1.8. Suggestions for Modifications of the IGD Group Therapy

The statements of the last main category were divided into two subcategories, one without and one with three further subcategories. A total of 43 codings could be assigned to the upper category including the patients’ views on reasonable group therapy modifications:

1. General conditions;
2. Conduction of group therapy;
  - Guidance by the group leader;
  - Content details;
  - Creation of tension.

In the first subcategory, three patients suggested additional individual therapy sessions, less content overlap with other therapy groups, and a closed group setting.

In the second subcategory, three patients proposed modifications related to consistent enforcement of group rules, well-structured preparation of sessions, and sufficient attention to all group participants. In addition, two patients indicated that they would generally like to work on the content in more detail by completing more tasks. Another patient wished topics to be more closely aligned with the needs of individual patients. To make the group more exciting, patients suggested the inclusion of more creative tasks, self-directed work, games, and discussions.

### 3.2. Interviews with Experts

The analysis of the expert interviews resulted in 151 codings that could be classified into six main categories. These are listed together with their subcategories and selected anchor examples in Table 4.

#### 3.2.1. Patient Requirements

The first main category included two subcategories with eight codings on patient requirements for the group therapy:

1. Indication;
2. Individual group ability.

In the first subcategory, the experts emphasize the importance of an appropriate indication for therapy before the start of the group. This includes a thorough diagnosis and the clarification of potential exclusion criteria. The group ability of the patient needs to be checked and a certain degree of motivation must be present. The content and structure of the group therapy should be explained to each patient in advance.

#### 3.2.2. Therapist Requirements

Nine codings were made in the second major category of requirements the professional group leader should fulfill. First, the therapist should be experienced and competent enough to respond to the group dynamics and to pay attention to all participants. Second, IGD-specific knowledge was considered mandatory and experience with/knowledge about computer games was considered as helpful.

#### 3.2.3. General Conditions

The third main category included a total of 58 codings in three subcategories with zero to four sub-subcategories:

1. Requirements of a functional group setting;
  - Group structure;
  - Group composition;
  - Task setting;
2. Therapy motivation;
  - Transparency;
  - Social competence;
  - Feedback and positive reinforcement;
  - Long-term consequences;
3. Presentation of contents.

Both experts said that structure was very important in group therapy. As many patients were lacking structure in daily life, structured therapy was helpful for a good, often first therapy experience. Each therapy session should consist of an opening phase, a main part, and a closing phase. Regarding group size, both experts agreed that a group of eight participants would be optimal. Furthermore, the experts argued that adolescents of different age groups were intentionally included in one group. Thereby older patients could be a role model for younger ones and take responsibility for them. This could lead to contrasting experiences to their everyday lives. However, different age groups would also create difficulties due to different levels of life experiences and maturity. This makes group rules even more important, and balanced age groups are desirable. Moreover, this subcategory contained statements about setting, dealing with different age groups and individual needs. The first expert would give the same tasks to all adolescents and add individual explanations. This is contrasted with the opinion of the second expert. She thinks that different, age-appropriate worksheets could be useful.

In the second subcategory, aspects for maintaining and increasing motivation to participate were subsumed in four further subcategories. The experts stated that motivational work started before the group program with the transparent presentation of the group concept, processes, and contents. Furthermore, motivation could be encouraged through social competence, feedback, and individual rewards for behavioral successes. One expert pointed out that an over- or under-challenging process would decrease motivation. Motivation can be increased by discussing long-term negative consequences of problematic gaming together. To illustrate these, pro and con arguments to maintaining the status quo could be collected.

The third subcategory summarized experts’ statements regarding the presentation of contents so that they could be integrated into the patients’ everyday lives in the best possible way. Both experts agreed that visualizing the content on a flipchart was sufficient. Moreover, a higher learning effect for the patients was assumed if they wrote down the discussed contents themselves.

#### 3.2.4. Reflection of the Therapy Contents

In the fourth main category, 51 codings could be classified from the data material into three subcategories and nine sub-subcategories:

1. Important/effective contents;
  - Psychoeducation;
  - Therapeutic diary;
  - Social competence training;
  - Emotion management;
  - Control mechanisms;
  - Building up alternative activities;
  - Reflection on the group participation and saying goodbye;
2. Challenging content;
  - Individual disorder model;
  - Situational control;
3. Scope for structuring the sessions.

Important and effective contents of the group therapy, as mentioned by the psychotherapists, were listed in the first subcategory and seven sub-subcategories. At the beginning of the group therapy, psychoeducation should take place. Problem awareness could be supported by helping patients to familiarize themselves with the diagnostic criteria of IGD and to consider which criterion applies to them. The vicious circle of IGD should help the participants to reflect on how the disorder emerged and persisted in their individual case. Regarding the most important psychoeducative contents, the experts mentioned the provision of information about long-term negative consequences of IGD and the explanation of gaming mechanisms that promote addiction. In addition, a therapeutic diary should be kept by the patients to encourage reflection on their usage times. The development of social competence was seen as especially important therapy content. According to the second expert, this could be practiced within the group setting. Moreover, patients’ deficits in perceiving and dealing with negative emotions should be addressed. Control mechanisms, especially in dealing with craving, should also be focused on. Furthermore, building up alternative, non-digital activities to gaming were indicated as essential. In the last group therapy session, patients should be guided to reflect on the past therapy weeks to appreciate the progress they had achieved. Prevention strategies for possible relapses were also named as a relevant aspect to be considered within the last group session. Finally, a farewell ritual should complete the group therapy, as many digital games have no end and saying goodbye is a rare topic in patients’ everyday life.

In the second subcategory, the experts referred to challenging therapy contents. Accordingly, the patients’ confrontation with personal stress factors and efforts to reduce habituated gaming behavior was described as demanding. Consequently, creating an individual disorder model including potential personal and social difficulties could lead to new psychological stress within the therapy group. Moreover, regaining control over gaming behavior was seen as difficult for the patients.

In the third subcategory one psychotherapist described the group content as comprehensive because sessions could be flexibly adapted and arranged with reference to the current group constellation and the clinical picture of IGD.

#### 3.2.5. Homework

The experts’ statements on homework in group therapy could be classified into three subcategories:

1. Relevance;
2. Motivation;
3. Therapeutic diaries.

One expert said that homework is particularly relevant for repetition and consolidation of therapy content.

How to maintain and increase motivation for homework among patients was listed in the second subcategory. One expert experienced that her patients did their homework regularly and responsibly if they felt that they identified with the group. In contrast to the first expert, the second expert found homework to be a difficult topic but considered using reminders as helpful. In addition, the motivation for homework could be increased by offering support and rewards.

The therapeutic diary was regarded as important in helping patients gain self-control through self-observation. One expert indicated that the diary should include information about the patient’s usage times, digital devices used, sleep times, alternative activities, and mood. It should be completed every day.

#### 3.2.6. The Role of Parents in Therapy

The last main category included statements about the role of parents in the therapy of their affected children.

According to the experts, involving the parents in therapy was difficult in clinical practice due to limited time and conditions. A parental group training on IGD parallel to their children’s group therapy was seen as highly valuable. Regardless of whether parents participate in parent training, they should definitely be present at a joint pre- and post-treatment consultation. In addition, one psychotherapist suggested that it might be helpful to talk to the parents about their child’s progress after half of the group therapy sessions were completed.

Overall, a positive effect on the therapy of the adolescents by actively involving their parents in the therapeutic process was emphasized by the experts.

## 4. Discussion

The aim of this qualitative interview study was to evaluate components of a group therapy program for adolescents with IGD and to identify requirements for potentially effective treatment approaches in clinical settings. The patients’ perspective was taken into account, as this is usually not the case in published studies of the field but of high information value. Additionally, professionals’ opinions complemented affected adolescents’ views by expert knowledge and profound clinical experiences.

According to a meta-analytic review, group therapy in general is an effective tool for children and adolescents, with no difference in effectiveness compared to individual psychotherapy [40]. At the same time, given the current lack of adequate intervention resources for the significant number of young patients with IGD, it is a highly economic tool where peer interaction can have unique therapeutic value [24]. Therefore, deeper insight into IGD-specific requirements for effective adolescents’ group therapy is urgently needed.

### 4.1. Patients’ Characteristics

Patients’ characteristics have been discussed repeatedly as a possible factor influencing the effectiveness of group therapy [23]. Among adolescent patients, age is the first factor that needs to be considered. According to the WHO, adolescence covers [41] the period from 10 to 19 years. Different levels of maturity require different approaches in interaction and content presentation, as the experts interviewed emphasized. However, according to the professionals, different age groups provide the opportunity, e.g., for older adolescents to act as role models and take responsibility for the younger patients. Based on the patients’ interviews, three core symptoms of IGD were highlighted: escaping stress and negative emotions, neglecting school and family obligations as well as other leisure activities, and losing control over gaming time. However, patients also described gaming as a safe place for trying out new roles and for experiencing positive peer interactions that cannot be obtained in real life. Two prominent aspects can be seen in the context of adolescent developmental tasks: identity formation and peer group integration. A recent prospective meta-analysis could identify problems in the attainment of developmental tasks including identity development and peer group membership to be associated with internalizing and externalizing psychological symptoms in a bidirectional way [42]. On the one hand, higher psychological symptoms predicted lower success and progress in the attainment of developmental tasks. On the other hand, failures with task attainment could be identified as risk factors for psychological symptoms. Hence, special attention should be paid to positive gaming experiences and adolescents’ attempts to achieve developmental tasks in the digital world. Taken together, perceived negative consequences of gaming might enhance change motivation, while positive associations with gaming most likely reduce change motivation if no alternatives are provided. Paulus et al. [6] identified low self-esteem, social isolation with poor interpersonal relationships and few or no real-life friends, impaired social functioning, and limited offline leisure activities as risk factors for developing and perpetuating IGD. Moreover, each of the patients interviewed suffered from mental comorbidities (most frequently, affective disorders and ADHD). A significant rate of comorbid symptoms in a clinical sample of adolescents with IGD was also described by Torres-Rodríguez et al. [43]. At this stage of research, only the PIPATIC program by Torres-Rodríguez et al. has placed a deliberate focus on the co-treatment of comorbidities [12] and could be shown to be superior to standard CBT [13]. Concordant with this finding, the experts of the current interview study stressed the consideration of comorbidities in conducting the group therapy, as they influence the patients’ abilities to follow group contents and to actively participate. Consequently, patients’ individual characteristics and needs should be targeted with regard to the content and setting of an IGD group intervention.

### 4.2. Group Content

The following therapy contents were defined as important and effective by patients and professionals and are supported by published studies on the treatment of IGD: psychoeducation [12,25,26,27,44], emotion management [12,25,27,44], behavioral analysis and modification (e.g., structuring daily routines, developing alternative activities, reduced usage times) [12,25,26,44], social skills training [25,28], and relapse prevention [26]. Furthermore, parents of affected adolescents should be actively involved in therapy [12,26,27,28]. This is in line with international opinions on the inclusion of parents as an important treatment component to address IGD-associated negative family aspects [45]. The experts of the current study stated that involving parents in the therapeutic process can have a positive impact on symptom reduction in affected adolescents by different mechanisms. Thus, our results are consistent with the findings of Lo et al.’s qualitative study where interviewed experts described family involvement as challenging but essential for successful interventions for internet addiction, including IGD [33]. For this, parents must learn to set appropriate limits and expectations. Positive intrafamilial communications and interactions should be promoted for better family functioning. Besides this, another aspect that might deserve attention comes from individual CBT with anxious children [46]. It assumes that parents’ therapy expectations influence therapy outcome in children and adolescents by modulating parental support and adherence. Moreover, an association between problematic family factors, such as parental psychopathologic burden and stress, and reduced active therapy participation of the child was assumed by the authors. Regular meetings with parents, in which adolescent group therapy contents are shared transparently and support offers are made, could be useful for this matter.

### 4.3. Group Setting

Besides the specific contents of an intervention program, both patients and experts of this study emphasized the importance of general conditions for a functional group setting. In current treatment studies, specifics for the setting of an IGD intervention program were usually not paid attention to [47].

The patients reported social skills and a sense of community to have an important role in a functional group setting. The latter (e.g., achieved through mutual exchange between patients) was also identified as one out of three effective treatment factors by the patients. This is in line with findings from a review that used a systematic PubMed search to discuss factors for effective group CBT in depressive adolescents [24]. Accordingly, a successful treatment outcome is associated with the presence of peers as a source of feedback, role model, and practice partner in dealing with symptoms and improving social skills. As adolescents with IGD often show reduced social competence [6], social skills training is a common component of IGD intervention programs and was defined as important by patients and experts. The experts also mention positive effects of interactive social skills training on therapy motivation in general. Together with a positive group atmosphere, this might make shy and socially insecure or anxious patients feel more comfortable in the group and trust their peers. This is the prerequisite for being able to be open, talk about difficulties, and try out new strategies. Correspondingly, most patients interviewed preferred a closed group setting rather than an open group setting in order to get to know and trust the other adolescents better. In (partial) inpatient settings this is often hard to realize.

Patients argued for the importance of a competent therapist leading the group intervention. Competent and well-trained therapists under effective supervision are considered significant in group interventions with addicted adolescents [48]. According to a meta-analysis including group CBT studies with children and adults, one of the therapist’s biggest challenges is to guide group cohesion to a focus on specific contents [49]. Based on the interview results of both patients and experts, this requires the setting to be continuously adjusted to the dynamics and needs of the group constellation. The selection, sequence and presentation of content could be adapted in this way, e.g., by flexible and varying work phases to increase therapy attachment and coherence.

Interviewed patients suggested the inclusion of more active elements in the group therapy, such as physical exercises and games to increase activity, excitement, and fun. Furthermore, patients and experts mentioned feedback and rewards for success as desired factors to increase motivation. This reflects elements of classic CBT and might be realized by, e.g., verbal reinforcers and token programs for accomplished tasks including therapeutic homework and conscientious keeping of gaming or mood diaries. Homework was considered to be important for the repetition and consolidation of therapy content by the interviewed experts and previous therapy studies [28,50]. By keeping a diary on mood, gaming and other activities, self-observation and self-awareness to gain situational control could be enhanced. However, since it requires special attention to problematic states and actions, building up of therapy adherence might be disturbed, especially within the first weeks [51]. This might be a reason why many patients of this interview study were not motivated to complete homework. Here, accompanying single individual therapy sessions (e.g., before the start of the group program, half the program, and after the last group session) might be beneficial. Moreover, inpatients reported that they would not see any benefit in completing the diary since their daily structure on the ward was highly structured and access to digital devices was limited. Hence, the form of the diary should be adapted to the individual situation, so that a personal benefit could be derived. In the view of the experts interviewed, identification with the group, reminders, therapeutic support, and rewards could support motivation and adherence. In their systematic review, Lee et al. [46] reported a moderate correlation between homework compliance and therapeutic relation in an individual CBT setting on anxiety disorders, as a further issue that might need to be considered.

Kaminer [48] listed seven crucial contextual factors for group therapies for addicted adolescents based on Eccles and Gootman’s [52] features to promote positive outcome of community programs on youth development:

1. Physical and psychological safety;
2. Appropriate structure;
3. Supportive relationships;
4. Opportunities for belonging;
5. Positive social norms;
6. Support for efficacy and mattering;
7. Opportunities for skill building.

These factors support major findings of the current study on the structural requirements for group therapy settings in adolescents with IGD and should be implemented regularly.

### 4.4. Limitations

The results of the study cannot be generalized and reflect individual opinions due to the qualitative study design with a small sample. The patient sample was heterogeneous regarding treatment duration and the overall treatment setting (day clinic or inpatient) at the time of the interview. It can be assumed that participants who had been in the treatment for at least eight weeks were more experienced with the program, which might have led to a more elaborate reflection on it. Although inpatients and patients at the day clinic attended the same IGD program and comparable therapies in general, the biggest difference between both groups was the time spent at home during treatment. Consequently, the possibility to transfer therapy contents to the home environment varied significantly. Thus, day-clinic patients had to take more personal responsibility for their gaming behaviors, which might have led to different views on the relevance of program topics. The minimum patient’s age was 12 and the majority of patients were 16 to 17 years old at the time of the treatment. Future studies should include a higher proportion of younger adolescents for a larger emphasis of their needs. As only one female patient was interviewed, the results were dominated by the experiences of male patients. The gender ratio for IGD patients is in line with the literature [53], but future studies should include more girls.

According to Mayring [54], high levels of agreement between the category systems of the analysis are difficult to achieve. Therefore, in the present study intercoder reliability was increased by revising the category systems by an independent rater.

The data were collected during the COVID-19 pandemic. Thus, mandatory contact restrictions had to be taken into account for the interview setting. Accordingly, a sitting distance of at least 1.5 m was maintained, and mouth-nose protection was worn by the interviewer and the interviewees at all times. Four interviews had to be conducted via telephone. These measurements might have created a personal distance between the interview partners. To preserve the highest possible naturalness of interaction, special care was taken to use encouraging and empathic communication techniques.

For reasons of economy and comparability a semi-structured interview guide was chosen. It was developed by clinical and scientific experts on IGD but, considering the novelty of this research field, had not been validated before. It can be assumed that different results would have been obtained by a higher proportion of inductive category-based evaluation of data material if the interview had been less structured and the interviewees had been encouraged to tell their stories more freely by asking more open questions. Further research comparing these different approaches should be encouraged.

Moreover, as this is a retrospective study, cognitive biases such as recall errors or memory bias must always be taken into account [55], especially for the patients who had been discharged for one to two years. Five out of the seven patients included were interviewed during their clinical treatment to minimize these biases. Finally, replying in a socially desirable manner is a common problem in interview studies [56]. This might have led to a bias towards less critical statements of the patients. To reduce this bias, the interviewer was independent of the patient’s treatment team and emphasized the confidentiality and anonymity of all information given.

Future studies on adolescent IGD programs are warranted that integrate and further evaluate aspects for effective outcomes based on individual, content- and structure-related aspects.

## 5. Conclusions

The present qualitative interview study is the first that has addressed patients’ and experts’ views on requirements of an effective IGD group therapy program in a clinical setting. Based on the participants’ responses, the following can be concluded:

IGD group therapy can serve as an effective tool to help affected adolescents in an efficient and useful way. On the one hand, relevant therapeutic content can be transmitted to more than one patient at a time, given that limited resources are a common problem. On the other hand, an environment can be built in which peers act as guided feedback providers, role models, and practice partners in dealing with symptoms, trying new functional behavior, and improving social skills. Potentially effective group intervention programs for adolescents with IGD should not only address up-to-date CBT-based therapy content (such as psychoeducation, daily structuring with alternative activities and reduced gaming times, emotion regulation and social skills training) and parental involvement. They also need to consider patient characteristics (including age and maturity, personal traits and comorbidities) as well as a functional group setting to create a motivating atmosphere in a safe, structured, appreciative, adaptable, and supportive environment.

The presented findings need to be viewed with caution, taking into account the small sample size and the non-representative sample structure. Future studies should, therefore, investigate the results in more detail and with larger sample sizes. The experiences of patients and psychotherapists could be of added value for the development and testing of effective IGD group programs in clinical care and research.

## Figures and Tables

**Table 1:** Patients’ characteristics.

	Age at the Time of Treatment	Gender	Treatment Duration at the Time of the Interview	Treatment Type	Comorbid Diagnoses
P1	17 years	male	8 weeks	day clinic	F32.1, F93.8
P2	12 years	male	4 weeks	day clinic	F32.1, F90.0, F91.3, F93.8
P3	16 years	male	10 weeks (discharged)	inpatient	F81.0, F90.0, F93.8
P4	17 years	male	14 weeks (discharged)	inpatient	F10.2, F12.1, F32.2
P5	16 years	female	4 weeks	inpatient	F93.8
P6	18 years (16 years)	male	4 weeks/2. stay (1. day clinic treatment in 2019 [12 weeks])	day clinic	F12.1, F33.2, F90.0, F93.8
P7	15 years	male	4 weeks	inpatient	F12.2, F15.1, F17.2, F32.2, F50.4, F90.0

Notes. P = patients; diagnoses were displayed with the specific ICD-10 code [38]; F10.2 = Mental and behavioral disorders due to use of alcohol: dependence syndrome; F12.1 = Mental and behavioral disorders due to use of cannabinoids: harmful use; F12.2 = Mental and behavioral disorders due to use of cannabinoids: dependence syndrome; F15.1 = Mental and behavioral disorders due to use of other stimulants, including caffeine: harmful use; F17.2 = Mental and behavioral disorders due to use of tobacco: dependence syndrome; F32.1 = Moderate depressive episode; F32.2 = Severe depressive episode without psychotic symptoms; F33.2 = Recurrent depressive disorder, current episode severe without psychotic symptoms; F50.4 = Overeating associated with other psychological disturbances; F81.0 = Specific reading disorder; F90.0 = Attention-deficit hyperactivity disorder (ADHD); F91.3 = Oppositional defiant disorder; F93.8 = Other childhood emotional disorders [38].

**Table 2:** Extracts from the patient interview guide (translation by the authors).

	Question
	Group therapy contents
1.	What brings you the most out of your treatment?
2.	When has the group been helpful for you? With what feeling do you leave a helpful session?
3.	What is less helpful/what is especially challenging for you in the group?
	Suggested alterations of the current group program
4.	Imagine you are a therapist, and you are leading the IGD group: what would you do differently?
	General group therapy setting
5.	How can the motivation for the group be maintained?
6.	What is particularly important for you regarding the group setting?
7.	How do you feel about the fact that new participants are joining the group?
	Dealing with homework
8.	What can increase the motivation to keep the diary continuously?

**Table 3:** Main categories and subcategories of the patient interviews with their anchor examples.

Main and Subcategories	Selected Anchor Examples *	Reference
1. Patients’ characteristics		
1.1 Escape	Escape from stress and problems at school or in the family:	
	“[I played digital games] because I just had a bunch of problems at home, at school and in my personal life.”	I. 06, l. 1276
	“[I’ve had] permanent trouble at home. And I just backed off because of that, [...] [I played] to just take my mind off of it.”	I. 05, l. 1026
	Escape from worries and difficult emotions:	
	“[..] I’m just gaming [...] because I have so many worries [...] because I’m depressed.”	I. 03, l. 491
	Escape from interpersonal problems: “I play games because I used to get bullied at school.”	I. 05, l. 1024
1.2 Neglect	Neglecting school and family responsibilities or leisure activities: “During school time I definitely neglected school. I didn’t go there at all […]. I’ve never done homework either way.”	I. 08, l. 1551
	“The biggest problem with that is that I pretty much neglect my family [...]. I also no longer engage in [other] recreational activities [than gaming].”	I. 03, l. 571–573
1.3 Loss of control	“[…] when I am gaming, I can’t stop that well.”	I. 09, l. 1715
2. Functional aspects of gaming	Being someone else: “I [could] be someone completely different [...] with a completely different story. And I could then open myself up and […]	I. 06, l. 1276
	lose myself in this world.” “[I game] because I can be a different person [there than I am in real life].”	I. 03, l. 493
3. General setting of the group therapy		
3.1 Requirements for a functional group setting		
3.1.1 Respectful interaction between group members	“I think it’s important that you get along with the people [...] and that you get along with each other and don’t trouble each other.”	I. 05, l. 1148
	“The […] [group members] don’t have to be best friends, but everyone has to get on well with each other. [It is important] that you […] follow the group rules. That is how it should be in a group.”	I. 06, l. 1350
3.1.2 Mutual support of group members	Support of restrained co-patients: “If you have a more open character yourself, you just try to include [the others] [...] in the group. And you always try to build up a little bond so that they are more familiar with the group itself and then some of them dare to say something in front of the group.”	I. 01, l. 136–138
	Of new group members: “I think you also have to be attentive to others, especially to the new […] [group members].”	I. 03, l. 661
	In the form of mutual encouragement: “When people [...] have accomplished something, what concerns the computer [use patterns], I also have the feeling that I have the motivation to achieve this. I am also happy for the person [for his success].”	I. 08, l. 1603
3.1.3 Stable group constellation		
3.1.3.1 Difficulty with unstable group constellation	Difficulty opening up in front of others: “I have so much social anxiety anyway and I don’t like to say anything in front of strangers. [...] Especially when talking about problems.”	I. 09, l. 1815–1817
	Composition of the therapy group of day clinic and ward: “[…] it’s actually quite difficult, because I was on the ward [...] and most of the […] other […] [group members] were from the day clinic. It is really difficult to get to know them personally.”	I. 06, l. 1354–1356
3.1.3.2 No difficulty with unstable group constellation	“I had no problems with adjusting to the new fellow patients in any way.”	I. 01, l. 134
	“[…] opening up to people, [...] I have no problem with that.”	I. 08, l. 1609
3.1.3.3 Positive group atmosphere	Getting to know new patients: “I think you have to get to know them first to really see “Yeah ok what’s he like? How should I behave?”	I. 03, l. 657
	Fun in the group: “If you can laugh with other people, you feel much more comfortable with them.”	I. 07, l. 1484
3.2 Working on tasks	Optimal way of task solving depends on the task: “It always varies. If it’s a task where you really have to work on it for yourself, then it’s good if you do it alone. If there’s a task where it’s about collecting a bunch of things, [...] then it’s cool if you do it in a group and one of you writes in front. Then there are also tasks where it’s good to work with a partner.”	I. 06, l. 1360
	Group work improves social competence: “[…] that you do more in the group. Because it also increases social competence, for example, which I would say from my own experience is usually less present in other gaming patients [...] That’s why I think that group work is best.”	I. 01, l. 148–150
	Working in groups of two: “I prefer to work in pairs.”	I. 09, l. 1825
	Form of the task working is unimportant: “I actually found it didn’t matter […]”	I. 05, l. 1176
3.3 Presentation of contents	Visual presentation of the content: “I am quite a [...] visual learner [...] the visualization of content is very important for me.”	I. 08, l. 1635–1637
	“I am rather a visual learner.”	I. 06, l. 1364
	Form of presentation of the content is unimportant: “[it plays] not a big role.”	I. 01, l. 158
4. Effective treatment factors		
4.1 Problem identification and discussion	“I think what was most helpful were the one-on-one sessions with the psychologists and psychiatrists. There we did not directly talk about my addiction, but also about the problems behind it […] why my gaming use became problematic.”	I. 06, l. 1294
4.2 Structure of everyday life	“That I create a daily plan, [...] so when my mom asks me to clean out the dishwasher, then I just do it […]. It no longer comes to conflicts because my mom thinks that I’m on the computer the whole time.”	I. 07, l. 1420
	“The most important thing for me is that this therapy has finally helped me to pursue my goals [...] all the things that I wanted to do all along, I have now started to do.”	I. 01, l. 58–60
4.3 Group cohesion	Exchange with other patients: “[…] to know that there are others…that you’re not the only one [...]. […] It’s also good to make progress with other people.”	I. 08, l. 1559–1561
5. Experiences with group therapy contents		
5.1 Level of knowledge regarding psychoeducation	Knowledge of IGD before the treatment: “I know what the risks are, what […] [gaming addiction] can do to you […]. But I already knew all that before.”	I. 07, l. 1388
	“I knew the risks of gaming before I went into treatment.”	I. 08, l. 1531
	Satisfaction with explanation about the disorder in group therapy: “In general, I felt very informed.”	I. 06, l. 1252
5.2 Helpful group content		
5.2.1 Psychoeducation	Understanding causes of pathological gaming: “[…] for example, [...] to learn about the triggers that tempt me to play.”	I. 01, l. 80
	Risk of developing addiction: “[to learn about] how quickly one can become addicted [to gaming].”	I. 05, l. 1126
	Negative effects: “What I just said at the beginning with the [negative] effects.”	I. 05, l. 1124
	Learning to deal with GD: “[to learn] how to deal with […] [gaming disorder] and what alternatives you have [...] that would be useful.”	I. 01, l. 80
5.2.2 Addressing individual problematic behavior	“To find another point of view or a new point of view or generally a point of view on one’s own addiction.”	I. 08, l. 1567
	“To find an alternative for it [gaming] so that you don’t fall back into this old pattern.”	I. 01, l. 74
5.2.3 Fun	“In this group you also have fun.”	I. 03, l. 597
5.2.4 Competent group leaders	“I found that it helped that Ms. X *[anonymized]* was always so nice to you and no matter what you said, she didn’t say “that’s wrong”, but also listened to everything and talked to you about it.”	I. 05, l. 1064
5.3 Interesting group content		
5.3.1 Origin of IGD and regaining control over gaming	“It was very interesting to talk about how much everyone played, what did they play, with […] [which device] did they play, with whom did they play [...]. And from that you could somehow also identify the reasons [for gaming addiction].”	I. 05, l. 1084–1086
	“This kind of self-control. [...] That you really pay attention “ok, I have this period now, I have a game and that’s it.”	I. 06, l. 1306–1308
5.3.2 Conception and mechanisms of games	“I think that’s the most interesting thing. [...] Seeing [what methods] game developers use to influence gamers’ brains to keep them engaged in the game.”	I. 08, l. 1577
5.4 Less helpful group content		
5.4.1 Contents without personal reference	“This often does not apply to me.”	I. 09, l. 1745
5.4.2 Content without gaming reference	“There were just [...] these non-gaming related modules, [...] which could have been done quite well in a skill group.”	I. 01, l. 88
5.4.3 Redundant content (with regard to other attended therapy groups)	“[Topics] that have already been discussed in another group. [...] were less useful.”	I. 01, l. 90
5.5 Challenging group content	First sessions in group therapy: “It was especially challenging that you should accept your own problematic behavior so quickly.”	I. 05, l. 1090
	“If you must be open in the group, then it’s quite a lot [...] but only at the beginning. At some point it was okay.”	I. 05, l. 1110–1114
6. Increase and maintenance of group therapy motivation		
6.1 Feedback of individual progress	“I think it’s always good when you see progress in yourself, because that’s when you realize “yeah ok, it’s getting better.” And then you have more motivation to stay on track.”	I. 06, l. 1342
6.2 Motivation through meaningful others	“I [came] to a new class. [...] And there was just a girl who I thought was cool. And at some point, I fell in love with her [...] And that was, among other things, a crucial point for me to really change and make something out of myself.”	I. 05, l. 1134
6.3 Positive reinforcement	“This Can-stop group [notes from the authors: a therapy group on substance use disorders], there you get a certificate when you [...] have achieved something. [...] To build in a reward system. [...] [that] could help quite a bit, I think.”	I. 08, l. 1593–1595
	“You have to see that the children [...] want to participate. And then say, for example, “Yes, okay, this week we’ll pay attention [and if] you are all doing well […] we can play something [notes from the authors: this suggestion refers to group games not digital games].”	I. 03, l. 623–625
6.4 Structuring of group therapy sessions	“ I would be motivated by more physical exercise during the group sessions.”	I. 01, l. 110
	“That you arouse the interest of the participants by providing more tasks, involving them more and simply bringing more fun into the group.”	I. 07, l. 1468
7. Therapeutic diary and homework		
7.1 Motivational aspects for diaries		
7.1.1 Self-responsibility	“I think that is a matter of the patient, how he motivates himself to fill in the diary.”	I. 01, l. 180
7.1.2 Professional support	“You just have to work more with the counselors I think, who then remind you every day to fill in the diary.”	I. 08, l. 1677
7.1.3 Diary structure	“The diary should be more easy to understand [...] then it would be easier and faster to enter the data.”	I. 03, l. 693–697
7.2 Volume of homework	“As it is now, it makes sense, but I wouldn’t add anything, because then at some point no one really wants to do it anymore.” “[If there would be more homework] I think it will be filled out even less, [...] so I would lack the motivation [to do the homework].”	I. 07, l. 1508 I. 09, l. 1845
7.3 Optional homework	“Especially at the beginning [...], I think it was extremely boring [...]. If you get one or two tasks that you should do until the next time, I don’t think it’s bad. [...] It doesn’t even have to be much, but just little things, if you’re really bored.”	I. 05, l. 1182–1186
8. Suggestions for modifications of the IGD group therapy		
8.1 General conditions	“[…] a little more time for the individual therapies.”	I. 01, l. 20
	“I think the group in itself was partly very similar to other groups that were done [...] with all the fellow patients.”	I. 01, l. 66
	“If everyone was from the ward for addictive disorders, then everything would be good. So, then it would be easier for me to open up in front of the others.”	I. 09, l. 1819
8.2 Conduction of group therapy		
8.2.1 Guidance by the group leader	“[…] that a better group climate is ensured and that the group leaders can be a bit more assertive [...] perhaps first of all make the group rules clear.”	I. 07, l. 1478–1480
	“Plan the group more and don’t just let the participants sit around bored.”	I. 07, l. 1478
	“The focus was a bit too much on one person [...] if the psychotherapist had paid a bit more attention to the group [...] it is often the case that somehow only two or three people in the group talk.”	I. 01, l. 28–44
8.2.2 Content details	“I found that some topics were not addressed enough. [...] It would be nice to discuss the topics in more detail.”	I. 05, l. 1118–1120
	“[…] the therapist should say “ok, we are now collecting topics that are very important to you”. [...] In this way you personalize it a bit more.”	I. 06, l. 1326
8.2.3 Creation of tension	“Maybe let the adolescents do more. [...] For example, give them more tasks[...] [or] let them do some handcrafts or whatever. Just do something that is also fun.”	I. 07, l. 1460–1462
	“Less paperwork, more discussion.”	I. 09, l. 1765
	“Maybe we should play a game every now and then [...] also with regard to the modules.”	I. 01, Z. 164

Notes. I. = Interview; l. = Line. * The original quotes were in German. Translation by the authors.

**Table 4:** Main categories and subcategories of the expert interviews with their anchor examples.

Main and Subcategories	Selected Anchor Examples *	Reference
1. Patient requirements		
1.1 Indication	“At first it is important to clarify whether the patient meets the diagnosis of internet gaming disorder. [...] This can be determined by means of self-assessment and external assessment questionnaires, which we give to the adolescents concerned and their parents. Self-assessment and external assessment are often very different, [which means that the affected adolescents often assess their usage behavior quite differently than their parents].”	I. 04, l. 724
	“Then it’s important to check if any exclusion criteria apply for participation in the group. For example, you must consider beforehand whether someone with Asperger’s autism can fit in and feel comfortable in the group. Sometimes there are also comorbid conditions that show up in problematic gaming and need special treatment.”	I. 04, l. 734
1.2 Individual group ability	Check for motivation to participate: “Before we accept a patient for the group, we have to check if there is enough motivation to participate in the group program. If not, we would have to do a lot of motivational work in the group and that would be difficult [as it would stop the rest of the group from progressing in the program].”	I. 04, l. 726
	“I would not offer the group program to a patient who absolutely resists it and sees no benefit in it.”	I. 02, l. 318
	Introduce group rules and make sure they are followed: “It is very important to present the group rules [to the patients] so that they know what to expect and what they have to stick to. This includes, for example, being on time and doing homework.”	I. 04, l. 730–732
2. Therapist requirements	Being experienced in working with groups: “You should always keep in mind that it’s not always about one individual, but that the group dynamic is also important […] that the group coherence is encouraged.”	I. 02, l. 330
	“You should know the comorbid disorders of all participants. [...] So that you don’t over- or under-challenge them.”	I. 04, l. 774
	Gaming and disorder-specific knowledge: “In addition to knowledge about gaming addiction, being a group therapist also requires you to keep up to date with computer games. You should definitely know from what age a game is allowed, what addictive factors it includes (e.g., the reward system) and what nicknames the patients have in the game. It is also advisable to simply try out certain games yourself.”	I. 04, l. 766–774
3. General conditions		
3.1 Requirements of a functional group setting		
3.1.1 Group structure	“Patients with internet gaming disorder often have no daily structure. Therefore, it is very important that a structured approach is taken in the group program.”	I. 02, l. 388
	“The beginning of each group session should always be similar. For example, the therapeutic diary should always be discussed at the beginning. Then there should be a main part in which knowledge is shared and self-reflection is encouraged. At the end, there should always be a final group discussion. This sequence gives patients a simple structure and they know what to expect in the group sessions.”	I. 04, l. 786–788
3.1.2 Group composition	Group size: “I accept a maximum of 8 patients in the group. This encourages patients to share experiences and create memories together.”	I. 02, l. 344
	Age differences: “I do not attach any importance to the age of the patients.”	I. 02, l. 348
	“It’s an advantage to have different age groups represented.”	I. 04, l. 790
	“I assign more responsibilities to the older adolescents in the group program.”	I. 04, l. 742
	“We have also noticed in our work again and again that it is difficult when you have, for example, 11-, 12-year-olds [...] and 16-, 17-year-olds [in one group]. And that’s why I think the group rules are very important.”	I. 04, l. 738–740
3.1.3 Task setting	“The same tasks are given to each patient. [...] [but to someone] with a severe ADHD, who is not medicated, I explain things differently [...] than to someone who is super shy. [...] That doesn’t just have to do with age. It also has to do with the individuality of the patient clientele.”	I. 02, l. 372–384
	“I would always do different worksheets. [...] The younger patients are often really overloaded with some tasks. Therefore, I make sure that the worksheets are easier to understand and the design is also a bit more appealing to them.”	I. 04, l. 744–746
3.2 Therapy motivation		
3.2.1 Transparency	“It is important to discuss the program goals and therapy content in detail at the beginning. Patients are often very grateful for this.”	I. 02, Z. 330
3.2.2 Social competence	“Then it is very important to get the patients to motivate each other. That’s why I also introduced this final round at the end of each group session, in which the patients assure each other that they will attending the next session. [...] Because then, of course, it is a social promise.”	I. 02, l. 338–340
3.2.3 Feedback and positive reinforcement	“Patients should always be encouraged to reflect on what has changed positively for them during the program. […] What has changed in family life? Are there perhaps fewer fights? What has changed in school?”	I. 04, l. 754–756
	“I also try to motivate patients with rewards.”	I. 04, l. 762
3.2.4 Long-term consequences	“I do not only [point out] the short-term negative consequences of internet gaming disorder, but also the long-term consequences. The patients usually don’t know about these.”	I. 04, l. 760
	“I always write a pro and con list of IGD to motivate patients to change. In doing so, I also consider short- and long-term consequences of gaming. [...]What is the argument for me to continue playing as usual? What is the argument against it, why should I stop excessive gaming?”	I. 04, l. 812
3.3 Presentation of contents	“I only use a flipchart. [...] I deliberately do it that way because in my group therapy and in my understanding of what is effective, it’s about getting in touch. [...] They are sitting in front of the screens all the time.”	I, 02, l. 392–396
	“I think it is enough [...] if you make a flipchart and try to visualize the contents nicely.”	I. 04, l. 880
4. Reflection of the therapy contents		
4.1 Important/effective contents		
4.1.1 Psychoeducation	“Of particular interest is the psychoeducational part of the program, in which the patients had to think for themselves whether the criteria of internet gaming disorder applied to them or not. [...] The self-knowledge that one is addicted is of course something completely different for the patient than when teachers or parents tell him that he is an addict. If it is possible to create a trusting, honest atmosphere in therapy and a patient recognizes for himself: “yes, I am on the verge of fulfilling such a diagnosis” [...], then an important requirement for successful treatment is met.”	I. 02, l. 234–238
	Psychoeducation content: “I would always do the addiction cycle, to explain how IGD develops in the first place. I think that’s important. The patients start to ask themselves “what is gaming actually doing to me? How did I actually start to become addictive?”	I. 04, l. 808
	“I have noticed that it is important to show the patients the long-term consequences of internet gaming disorder. They should think for themselves what it would be like if they didn’t graduate from school because of excessive gaming, if they were constantly dependent on the state, on their parents...that they couldn’t lead an independent life. They often don’t want that to happen.”	I. 04, l. 836–842
	“I also think it’s important to explain the game mechanisms to the patients. [...] They should know the addictive characteristics of games and how game developers deliberately use them to achieve high user retention. Patients should also understand the reward system used in digital games. Most of them are not aware of it at all.”	I. 04, l. 810
4.1.2 Therapeutic diary	“In the therapeutic diary, patients can record how much they played at the beginning of therapy and how that changed after therapy.”	I. 04, l. 816
4.1.3 Social competence training	“The social competence training [...] is very important to encourage patients to think about whether they behave toward others the way they want to.”	I. 02, l. 238–240
4.1.4 Emotion management	“I always discuss with patients how to deal with stress and negative emotions [...]. “How can I deal with anger? How can I deal with grief?” I find these are the most difficult emotions for adolescents. They often have to learn new strategies for dealing with these emotions. For example, they don’t know what to do when they’re angry, and in the past, they’ve then often turned on the computer.”	I. 04, l. 828–830
4.1.5 Control mechanisms	“You should consider with the patient what technical settings and rules are necessary to keep gaming under control. The patient could, for example, set certain gaming times or agree not to play until homework has been completed.”	I. 04, l. 822–826
4.1.6 Building up alternative activities	“The patients have to find new alternative activities and hobbies they like, because when the patients reduce their gaming time, they should have alternative activities they would like to engage in [...]”	I. 04, l. 814
4.1.7 Reflection on the group participation and farewell	Retrospective: “At the end of therapy, a proper farewell ritual is very important. Patients should realize that they have now spent eight weeks or eight sessions together. Social cohesion is an important aspect of the group program. The patients should also look back at the beginning of therapy. It is often the case that at the beginning none of the participants wanted to take part in the group and now they have managed to do so [...].”	I. 02, l. 248
	Farewell: “The last session is very important. [...] To understand what it means to say goodbye. Because that is something that is rarely the case in computer games. [...] Somehow goodbye never happens because you just go off or change the game. And in everyday life, in everyday social life, it’s also rarely about saying goodbye to each other properly.”	I. 02, l. 242–246
	Relapse prevention: “Relapse prevention should be addressed at the end of the course. [...] The early warning signs of relapse and risk situations should be written down [...]. An emergency kit should be packed. Patients should determine who to contact if they relapse.”	I. 04, l. 830–832
4.2 Challenging content		
4.2.1 Individual disorder model	“Patients should work out an individual disorder model for themselves [...]. In this way, they become aware of the personal and external factors that have led them to excessive gaming. The challenging part is that the patients have to admit their weaknesses. It is often stressful for them to realize what they are actually trying to suppress by gaming.”	I. 04, l. 866–872
	“Family stressors should also be worked out. It should be talked about whether the parents have separated, or whether there was a lot of arguing, or whether there was violence at home.”	I. 04, l. 874
4.2.2 Situational control	“I think the module where it’s about exercising situational control is very difficult. Because you often realize that a lot of patients have never reflected on their high usage times and what comes with it.”	I. 02, l. 270–282
4.3 Scope for structuring the sessions	“The modules leave room for flexibility. [...] You can choose modules based on what’s relevant to patients at that moment.”	I. 02, l. 286–292
	“To stick strictly to the module is not possible with this topic anyway. [...] It requires a therapist who is also experienced enough to respond flexibly to the needs [of the patients] at that moment.”	I. 02, l. 306
5. Homework		
5.1 Relevance	“I believe that the homework is very important [...]. At the beginning of the session, the homework of the last week should be discussed. I also ask the participants to briefly recall the contents of the last session.”	I. 04, l. 898–900
5.2 Motivation	“I think that for adolescents who have a rather negative attitude towards homework, they do it very well. [...] So, I think it’s going quite well.”	I. 02, l. 414–416
	“Homework is always a difficult topic. I think it’s important to remind the patients to do it regularly.”	I. 04, l. 940
5.3 Therapeutic diary	“It’s important to explain to patients why keeping a therapeutic diary is important. The advantage they gain from self-observation must be made clear to them. They should realize what effect self-observation has on their own behavior.”	I. 02, l. 428
	“The therapeutic diary makes patients aware of everything they have done on the Internet and how long they have been doing it. This includes not only the time spent on the computer, but also the time spent on the smartphone. They can also enter in the diary when they went to bed and how long they slept. They can also note what alternative activities they did and what their mood was while doing them.”	I. 04, l. 924–936
6. The role of parents in therapy	“If you could include the parents, it would be nice. [...] But yes, that is difficult, because the parents are usually only involved in the individual setting or in family sessions.”	I. 04, l. 904–906
	“I would like it if the parents were in a “parent training” at the same time, because then I think the patients see that the parents are also doing something [...]. For the parents it is simply important to know how they can support their children at home. [...] A parent group program would be more intensive than if they now only go to a family session every three weeks.”	I. 04, l. 912–916
	“When half of the sessions are completed, it is important to ask the parents if anything has changed at home. Their feedback on the course of therapy is very important.”	I. 04, l. 920

Notes. I. = Interview; l. = Line. * The original quotes were in German. Translation by the authors.

## Data Availability

The data presented in this study are available on reasonable request from the corresponding author.

## Appendix A

**Table A1:** Patient interview guide (translation by the authors).

	Question
	Current state of knowledge of psychoeducation and individual gaming behavior
1.	At this point in your treatment, how informed do you feel about your use of the Internet and online games? 1.1 What could be discussed more intensively? 1.2 What is too much?
2.	What do you think is your biggest problem in terms of online gaming?
	Group therapy contents
3.	What brings you the most out of your treatment?
4.	When has the group been helpful for you? With what feeling do you leave a helpful session?
5.	What content of the IGD group do you consider most interesting?
6.	What is less helpful/challenging for you in the IGD group?
	Suggested changes/additions to the current therapy
7.	Imagine you are a therapist, and you are leading the IGD group: what would you do in a different way? 7.1 What content would you add?
8.	How did you achieve a lasting change in your behavior and reduce your gaming?
	General therapy setting
9.	How can the motivation for the IGD group be maintained?
10.	What is particularly important for you regarding the group setting?
11.	How do you feel about the fact that new participants keep joining the IGD group? 11.1 Can you think of anything that might make it easier to be more open in front of other patients?
12.	How do you prefer to work on the tasks in the IGD group (alone, in groups of two, group discussion)?
13.	What is the best way for you to present the group’s content (flipchart/whiteboard)? Can you think of any improvements in this regard?
	Dealing with homework
14.	What can increase the motivation to keep the diary continuously?
15.	Let’s assume that the diary would become more extensive, for example, by including information on the daily structure. How realistic is it that it will still be kept regularly?
16.	Is there anything else important you want to talk about that I haven’t asked yet?

## Appendix B

**Table A2:** Expert interview guide (translation by the authors).

	Question
	Requirements for participants
1.	How is a good selection of participants made? (Indication, group ability)
2.	How can the age difference between patients be handled in the group therapy setting?
3.	What can maintain motivation to participate in group therapy?
	Requirements for group leaders
4.	What are the requirements for the group leader?
	General therapy settings
5.	What is important to consider regarding the group setting?
	Group therapy contents
6.	What content should be included in a group program?
7.	What is effective for participants in group therapy? How is this noticeable?
8.	What is challenging for participants in group therapy? How does this become noticeable?
	Presentation of the contents
9.	What form of presentation of the content addresses the patients well? (Reference: age differences of the participants) 9.1 What improvements can you think of for the presentation? 9.2 How can the content be integrated into everyday life in a more lasting way?
10.	What aspects should be included in an Internet-diary to best encourage reflection on Internet use?
11.	What is your experience with homework? Which form is best for it?
12.	Is there anything else important you want to talk about that I haven’t asked yet?
